# Isolate Circulating Mesenchymal Stromal Cells Without Growth Factor Administration and Using Density Gradient

**DOI:** 10.1155/sci/5545892

**Published:** 2025-06-19

**Authors:** Jason Ma, Chung-Chuan Hsiung, Tzu-Hsien Yang, Hsiu-Yen Sun, Ming-Ling Kuo

**Affiliations:** ^1^Department of Microbiology and Immunology, Graduate Institute of Biomedical Sciences, College of Medicine, Chang Gung University, Taoyuan, Taiwan (ROC); ^2^Department of Medical Trial, Bo-ai Clinic, Kaohsiung, Taiwan (ROC); ^3^Division of Allergy, Asthma and Rheumatology, Department of Pediatrics, Chang Gung Memorial Hospital at Linkou, Taoyuan, Taiwan (ROC); ^4^Department of Pediatrics, New Taipei Municipal TuCheng Hospital, New Taipei, Taiwan (ROC); ^5^Center for Molecular and Clinical Immunology, College of Medicine, Chang Gung University, Taoyuan, Taiwan (ROC)

**Keywords:** differentiation, immunomodulatory abilities, mesenchymal stromal cells, peripheral blood

## Abstract

Mesenchymal stromal cells (MSCs) are recognized for their differentiation and immune regulation capabilities, which enhance their potential for treating various diseases. MSCs can be sourced from diverse tissues, with peripheral blood (PB) serving as a viable alternative to bone marrow. We now present an alternative strategy that eliminates the need for preadministering growth factors, utilizing density gradient methods, and culturing target cells in medium supplemented with autologous serum. PB was collected through venipuncture and then coincubated with glycerin. After incubation, a thin layer of cells above the red blood cells (RBCs) was isolated, showing an increased population of CD34^−^CD45^−^ cells compared to PB mononuclear cell (PBMC) isolation using Ficoll gradient. After culture, adherent spindle-shaped cells were identified and collected to assess MSC surface markers, demonstrating their differentiation potential into adipocytes, osteocytes, and chondrocytes, thus, fulfilling the criteria for MSCs. The population doubling time (PDT) of isolated PB-MSCs was approximately 30–40 h in early passages. These PB-MSCs also exhibited immunomodulatory functions and are capable of suppressing T cell activation. We believe this protocol supports PB as a convenient alternative source for MSC isolation and offers new strategies for acquiring and maintaining PB-MSCs.

## 1. Introduction

Mesenchymal stromal cells (MSCs), as defined by the International Society for Cellular Therapy (ISCT), are plastic-adherent cells that express the surface markers CD73, CD90, and CD105, lack hematopoietic and endothelial markers, and can differentiate into adipocytes, chondrocytes, and osteoblasts [1]. The multiple differentiation potentials of MSCs increase their capacity for tissue regeneration and their immunomodulatory abilities broaden their clinical applications [2–6]. MSCs engage with immune cells and participate in both innate and adaptive immunity through cell-to-cell contact or paracrine signaling [7]. Notably, MSCs have been shown to suppress adaptive immune responses directly by inhibiting T cell proliferation and indirectly by modulating the antigen presentation of dendritic cells [8]. Furthermore, the suppressive ability of MSCs on activated T cells has evolved into concepts of quality control after MSCs are isolated and expanded using in vitro methods [9, 10].

MSCs are sourced from tissues like bone marrow, adipose tissue, and umbilical cord [11]. Though numerous clinical trials focus on bone marrow-derived MSCs (BM-MSCs) [12], bone marrow aspiration is a painful procedure [13], causing isolating MSCs from other reservoirs more appealing. It has been reported that in human peripheral blood mononuclear cells (PBMCs), a small population of CD34^−^ mononuclear cell (<1%) have adherent and self-proliferation ability, which is later defined as blood mesenchymal precursor cells (BMPCs) or circulating MSCs [14]. These cells show similar characteristic with BM-MSCs, only differ in CD73 expression level [15]. Following studies demonstrated that MSCs proportion is around one in 10,000 bone marrow mononuclear cells [16], while only one in a hundred million of PBMCs [17]. To increase the isolation possibility and decrease required blood amounts, researchers use density gradient and growth factors such as granulocyte colony-stimulating factor (G-CSF) to increase MSCs isolation rate [18]. Still, there are some discrepancies in this procedure. The administration of G-CSF is a time-inconvenient procedure with side effects such as headache, vomiting, nausea, and muscle pain [19]. G-CSF may also exacerbate sterile inflammation and autoimmune diseases by neutrophil-dependent tissue damage [20]. Nevertheless, although density gradients can select specific cell populations, the process of removing the density reagent may decrease or even eliminate the yield of target cells or rare cells [21]. Therefore, the development of isolation procedure without G-CSF priming and the use of density gradient may provide advantages to further clinical applications of peripheral blood (PB)-MSCs.

In this study, we demonstrate an alternative procedure that can obtain more CD34^−^CD45^−^ cells after venipuncture. Additionally, MSCs were successfully isolated from the PB of two out of six individuals without density gradient and growth factor administration. These MSCs performed well differentiation abilities and immunomodulatory functions in humanized culture conditions. We believe that this protocol may provide new strategies for obtaining and maintaining PB-MSCs.

## 2. Materials and Methods

### 2.1. Collection of Human Blood Samples

Adult PB was obtained from 14 healthy volunteers, including eight males and six females, in accordance with the approval of Chang Gung Medical Foundation Institutional Review Board (Approval No. 202100134A3/2101250066). The informed consent was signed by each subject participated in this study.

### 2.2. Isolating MSCs in PB

**Figure 1:**
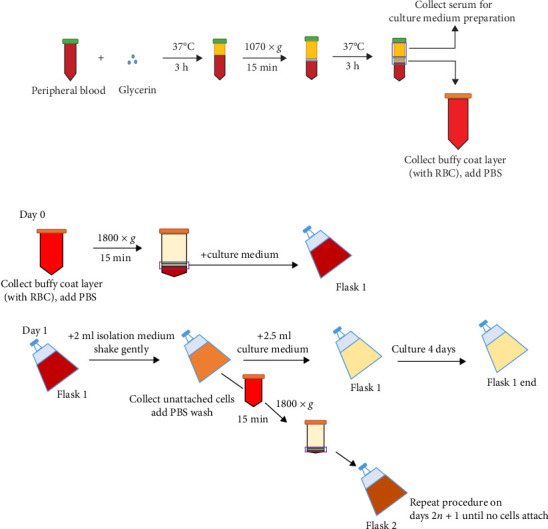
Brief diagram of glycerin incubation protocol for PB-MSCs candidate isolation. PB was collected and mixed with glycerin. This mixture was then incubated at 37°C, centrifuged, then continue incubated at 37°C. Serum was collected for culture medium preparation, while buffy coat layer with partial RBCs was collected and transferred to new flasks for PB-MSCs candidate isolation. This procedure was repeated every 2 days until no attach cells were observed. (A) Brief process for collecting buffy coat layer and serum. (B) Brief process for collecting attached PB-MSC candidates.

The isolation protocol was performed according to the description in Taiwan Patent No. I656215 (2019), as the glycerin incubation protocol (Figure 1). For isolation, the use of 50 ml adult PB usually grants higher chance to establish MSC cultures. Each PB sample was collected with vacutainers containing sodium heparin, and then, mixed without or with glycerin, for regular PBMC collection with Ficoll–Plaque (GE Healthcare) density gradient or glycerin incubation protocol, respectively. For the glycerin incubation protocol, PB was incubated at 37°C for 3 h followed with the centrifugation at 1070 × *g* for 15 min. This procedure was repeated for two to three times. The plasma were collected from the top layer, decomplementized (56°C, 30 min), filtered with 0.22 and 0.10 μM filters, and used for future cultures. A thin layer of cells laying above red blood cells (RBCs) was collected and washed with phosphate-buffered saline (PBS). These cells were centrifuged with 1800 × *g* for 15 min. This step was repeated until most RBCs were removed, normally for three rounds. Candidate cells for MSC isolation were then resuspended and cultured with MSCs culture medium (MCM), which is composed with keratinocyte-serum free medium (Gibco) supplemented with 20% of donor plasma, 50 μM of L-ascorbic acid 2-phosphate sesquimagnesium salt hydrate (Sigma), 18.4 μM of N-Acetyl-L-cysteine (Sigma), and 1% of PLTMax (Mill Creek Life Science). After 24 h, unattached cells were collected, centrifuged to remove cell debris, transferred, and cultured in a new flask for 48 h. The attached cells were cultured for 4 days, then, detached by TrypLE (Gibco), and transfer to a new flask to determine whether these cells can be reattached. This procedure terminates while no attach cells can be further obtained (brief protocol shown in Figure 1B).

### 2.3. Surface Marker Profiling for MSCs

Cells were stained with CD45-PerCP (eBioscience) and CD34-PE (Biolegend) for population analysis in PB, while CD11b-PE (TONBO biosciences), CD14-FITC (eBioscience), CD19-FITC (eBioscience), CD34-FITC (eBioscience), CD45-FITC (Invitrogen), HLA-DR-FITC (eBioscience), CD90-Percp-Cy50.5 (eBioscience), CD73-APC (eBioscience), CD105-PE-Cy7 (eBioscience), CD44-eFlour 450 (eBioscience), and CD166-PE (eBioscience) were used for characterizing candidate cells as MSCs. After staining, cells were washed with 1 × PBS. The fluorescent signals on cells were analyzed by Attune NxT flow cytometer and its software (Thermo).

### 2.4. The Confirmation of Differentiation Potential of Isolated MSCs

Isolated MSCs (1 × 10^4^) were seeded in 24-well culture plates with MCM until cells were attached. Cells were then incubated with appropriate differential medium for 14 days. For adipogenesis differentiation, 0.5 mM 3-isobutyl-1-methylxanthine (IBMX; Sigma), 1 mM insulin (Thermo), 60 μM indomethacin (Sigma), and 1 μM dexamethasone was supplemented in MCM. The osteogenesis differentiation kit (STEMPRO, Gibco) or chondrogenesis differentiation kit (Gibco) was also applied to differentiate MSCs into osteocytes or chondrocytes. Half of the culture medium was refreshed twice a week, until cells were fixed by PBS–based 4% paraformaldehyde after differentiation. Adipocytes were stained with oil red O (Sigma), while osteocytes were stained with Alizarin Red S (Sigma) and chondrocytes were stained with Alcian Blue (Merck) for 1 h to confirm MSC's differentiation potential. Microscopic results were examined with Olympus IX51 inverted fluorescent microscope and the pictures were taken with Olympus DP21 system.

### 2.5. Calculation of Population Doubling Time (PDT)

PDT was calculated according to the equation: PDT = culture time (CT)/population doubling number (PDN). PDN was calculated according to the formula: PDN = log *N*/*N*_0_ × 3.31, where *N* represent the cell number at the end of the culture and *N*_0_ represent the cell number at the beginning of culture. Cells were plated 3 × 10^4^ cells in 25 cm^2^ culture flasks until reaching confluence. Culture medium was exchanged after 3 days of culture if necessary. The cells were detached by TrypLE (Gibco) and counted for the calculation of PDT.

### 2.6. Cell Viability Assay (CCK-8) for Glycerin Cultured Stem Cells

PB-MSCs were incubated in a glycerin-containing culture medium or in a control medium at 37°C in a humidified atmosphere of 5% CO_2_ for 3 h. Following incubation, the cells were transferred to a 12-well plate and cultured for 3 days. Then, the cells were washed with PBS and a CCK-8 assay (Biotools) was performed according to the manufacturer's instructions.

### 2.7. Colony Forming Ability and Crystal Violet Staining Assay

Cells were seeded at 37°C in 5% humidified CO_2_ with the density of 100 cells/cm^2^ in six-well plate and cultured for 14 days. After culture, cells were washed with PBS and fixed with 4% paraformaldehyde for 30 min. Cells were then washed and stained with 0.05% crystal violet in methanol for 20 min at room temperature, then washed with ddH_2_O for capture.

### 2.8. RNA Isolation and Quantitative Real-Time PCR Analysis

Total RNA was extracted from isolated undifferentiated or differentiated PB-MSCs using TRIzol reagent (Invitrogen). The cDNA was generated using oligo-dT primers (Thermo) and M-MLV reverse transcriptase (Invitrogen). Real-time PCR was performed using SYBR Green Master Mix (Thermo) and amplified by the CFX Connect Real-Time PCR system (Bio-Rad). Real-time PCR reaction conditions were 95°C for 10 min then 40 cycles of 95°C for 15 s and 60°C for 1 min. The relative expression of each gene was calculated by normalizing the levels to the expression of HLA-β2 m, while the fold change was relative to undifferentiated cells. Primers are listed as Table S1.

### 2.9. PBMCs Isolation and Immunomodulation Assay for MSCs

Candidate cells and PBMCs isolated by Ficoll–Plaque were collected after 10 ml of adult PB was collected by venipuncture. RBCs were lysed and 2 × 10^6^ cells were prepared for CD34^−^CD45^−^ cell population analysis by flow cytometry. To examine the immunomodulation ability of MSCs, 1–8 × 10^4^ MSCs were preseeded in 96-well plate with RPMI medium supplemented with 20% of human serum according to different MSC to T cell ratio (from 1:2.5 to 1:20). CD4^+^ T cells were then positively selected from PBMCs with MACS system (Miltenyi Biotec) and further stained with cell proliferation dye eFlour670 (Thermo). These T cells were activated by anti-CD2, anti-CD3 and anti-CD28 conjugated activation beads, which was provided by human T cell activation/expansion kit (Miltenyi Biotec). The activation beads to cell ratio was 1–2 and cells were cocultured with MSCs for 4 days. The proliferation rate was analyzed by flow cytometry and indicated as immunomodulation ability of MSCs.

### 2.10. Enzyme-Linked Immunosorbent Assay (ELISA)

After T cell activation, culture supernatants were collected and subjected to the ELISA for the concentration of IL-2 or IFN-γ (BD Biosciences), followed with manufacturer's instruction. Absorbance was measured by ELISA reader at 450 nm (Bio-Rad).

### 2.11. Statistical Analysis

Results are presented as mean ± SEM. Significance was assessed with two-tailed *p*-Value calculated Mann–Whitney *t* test for nonparametric distribution or nonparametric one-way ANOVA test with Kruskal–Wallis test for multiple comparisons. *p*  < 0.05 is considered significant. All graphs and statistical analysis were generated and performed using GraphPad Prism 9.0 software.

## 3. Results

### 3.1. Isolating CD34^−^CD45 Cells in PB

**Figure 2:**
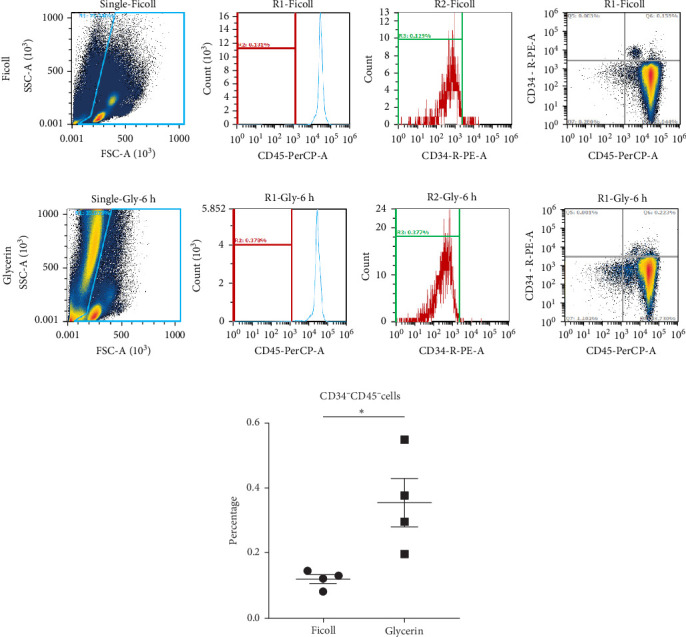
Population of CD34^−^CD45^−^ cells are enriched after glycerin incubation. PB was collected by venipuncture with a total volume of 10 ml. The population of CD34^−^CD45^−^ in (A) PBMCs isolated with Ficoll–Plaque density gradient. (B) Target cell layer isolated from glycerin and heparin incubated PB was detected by flow cytometry. (C) The percentages of CD34^−^CD45^−^ cells in total cells. Data are presented with dot plots or histograms and the percentages of CD34^−^CD45^−^ cells are shown as mean ± SEM (*n* = 4). *⁣*^*∗*^*p*  < 0.05 is demonstrated with nonparametric Mann–Whitney *t* test.

**Figure 3:**
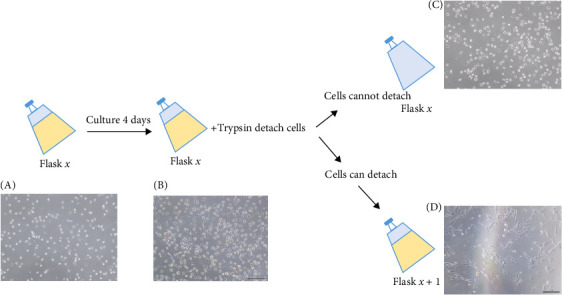
Isolation profile of mesenchymal stromal cell candidates. PB was collected by venipuncture with a total volume of 50 ml. Target cell layer isolated from glycerin and heparin incubated PB was collected and cultured with MCM. (A) Approximately 7 days after isolation, (B) cells were cultured for 4 days, (C) sphere cells that couldnot detach, and (D) detached spindle cells reattach in new flask. Representative images were taken by phase contrast microscope with 40x magnification.

Since MSCs are characterized as CD34^−^CD45^−^ adherent cells in PBMCs and that they occur in very low frequency, we tried to augment the yield of CD34^−^CD45^−^ cells based on the protocol shown in Figure 1. The results demonstrated that this isolation method can capture approximately three times more CD34^−^CD45^−^ cells after culturing PB with glycerin compare with PBMCs isolated with Ficoll–Plaque isolation method (Figure 2A). The PB from six individuals was further collected to determine whether more CD34^−^CD45^−^ cells were isolated with this method. With the culture protocol shown in Figure 1B, the isolated CD34^−^CD45^−^ cells were cultured until they were attached to the flasks. We found that cells from all six individuals were able to attach to the plastic surface approximately around day 7, with two distinct cell morphologies (sphere and spindle shapes, Figure 3A,B). Both types of cells slowly proliferated and the cells were enlarged. The sphere cells could not be detached by TrypLE (Figure 3C). However, detached spindle shape cells were able to reattach and then proliferate. There cells were considered as MSC candidates (Figure 3D). With further 3–6-day culture, 1–2 × 10^6^ MSC candidates were obtained for MSCs characteristic determination. Cells can be maintained with one-fifth ratio dilution for 10 further passages before cells stopped to proliferate.

### 3.2. Characterizing PB-MSC Candidates

**Figure 4:**
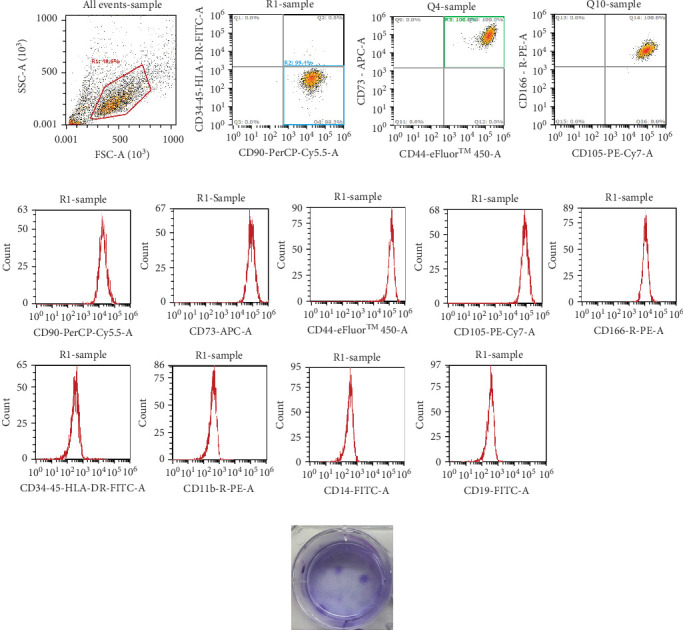
Isolated cells preform MSCs surface markers and colony formation ability. Isolated cells were labeled with CD34, CD45, HLA-DR, CD11b, CD14, CD19, CD90, CD73, CD105, CD44, and CD166 for MSC profiling and analyzed by flow cytometry. (A) Cell composition and (B) shows fluorescent intensity of each marker. Isolated cells all preform MSC-related surface markers. Representative data are presented with dot plots with four quadrant diagram or histograms of each cell surface marker. (C) Demonstrate colony formation ability by staining colonies with 0.05% crystal violet after culture.

**Figure 5:**
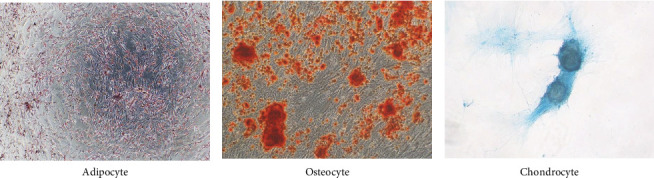
The differentiation potential of adipogenic, osteogenic, and chondrogenic of isolated cells. Cells detached from previous culture were cultured with differentiation medium for 14 days. (A) Cells cultured with adipogenesis differentiation medium and stained with oil red O solution. (B) Cell cultured with osteogenesis differentiation medium and stained with Alizarin red solution. (C) Cells cultured with chondrogenesis differentiation medium and stained with Alcian blue solution. All isolated cells were able to differentiate into three types of cells. Representative images for multilineage potential staining were taken by phase contrast microscope with 40× magnification.

**Figure 6:**
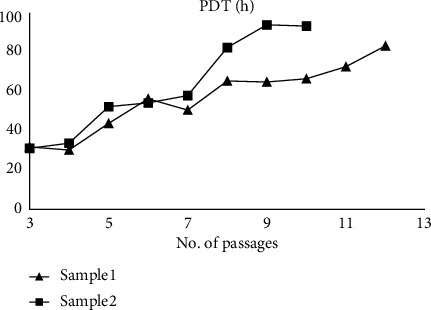
The population doubling time of PB-MSCs over passages. PB-MSCs were cultured at a density of 3 × 10^4^ cells in 25 cm^2^ culture flasks from the start of the culture. Once the cells reached confluence, they were detached using TrypLE, and cell numbers were counted to determine population doubling time (PDT). Results were presented as line graphs for each isolated cell line.

To confirm whether these candidates are MSCs, the cells were detached and stained with MSC surface markers, then subjected with flow cytometric analyses. These cells preformed <1% of CD11b, CD14, CD19, CD34, CD45, and HLA-DR negative, while >99% of cells were CD90, CD73, CD105, CD166, and CD44 positive, which fulfill ISCT's MSC definition (Figure 4). Stemness-related genes, *CXCR4*, *LGR5*, NANOG, *and NESTIN*, are also expressed in these candidate cells (Figure S1), while these cells also successfully differentiated into adipocytes, osteocytes or chondrocytes with 14-day differentiation procedure, suggesting that these cells are qualified for ISCTs MSC characteristics (Figure 5 and Table S2). Finally, the PDT for PB-MSCs were around 31 h, and slowly increase to more than 60 h after 8 passages (Figure 6).

### 3.3. Immunomodulatory Function of PB-MSCs

**Figure 7:**
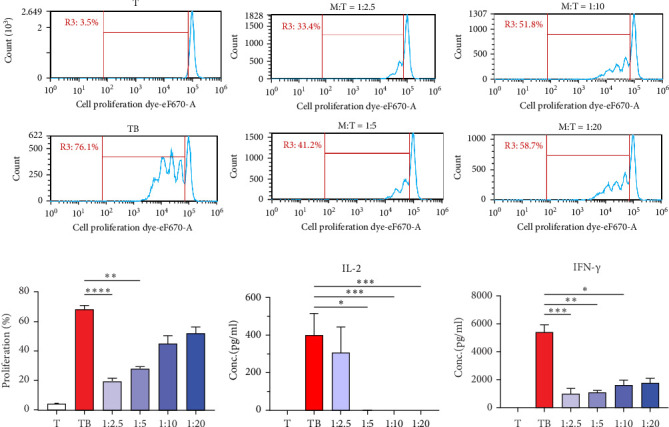
Isolated MSCs demonstrate immunomodulatory effects. MACS bead-activated CD4^+^ T cells stained with cell proliferation dye eF670 were cocultured with isolated MSCs for 4 days. (A) Represent the dividing pattern of CD4^+^ T cells cocultured with different numbers of MSCs. (B) Demonstrate the percentage of dividing CD4^+^ T cells, (C) IL-2, and (D) IFN-γ concentration in culture supernatants. Isolated PB-MSCs suppressed the proliferation of CD4^+^ T cells and downregulates IL-2 and IFN-γ. Data are presented by distribution chats or histogram. The percentage of dividing responder cells, IL-2 concentration or IFN-γ concentration was shown by mean ± SEM (*n* = 7). *⁣*^*∗*^*p* < 0.05, *⁣*^*∗∗*^*p* < 0.01, *⁣*^*∗∗∗*^*p* < 0.001, and *⁣*^*∗∗∗∗*^*p* < 0.0001 with nonparametric one-way ANOVA test together with Kruskal–Wallis test for multiple comparisons.

To examine whether these PB-MSCs have immunomodulatory functions, a humanized in vitro T cell activation system was established. The CD4^+^ T cells were isolated from PBMCs, activated with anti-CD2, anti-CD3, and anti-CD28 activation beads according to the manufacturer's instructions and cultured with human serum to mimic T cell activation within each individual. To our results, T cell proliferation is arrested while culturing MSCs together with activated T cells from 1:2.5 to 1:20 (MSC to T ratio), whereas T cells nearly stopped proliferating below the ratio of 1:5. Additionally, the production of IL-2 and IFN-γ are also suppressed, implying that isolated MSCs remains immunomodulatory function (Figure 7). Our results indicated that PB-MSCs collected from this procedure remains capability for differentiation and immunomodulatory ability.

## 4. Discussion

Isolation methods of MSCs from different tissue residues have been springing up for the past decade. Even though fibroblast colony formation cells in PB were first mentioned by Friedenstein in 1971 [22], it is continuously debated whether these cells exist until 21st century, whereas these cells started to be isolated from large amount of blood [10] or after G-CSF priming [14]. However, side-effects caused by G-CSF may affect donors' will, while it is also reported that priming with G-CSF would decrease the expansion potential and have lower prostaglandin E2 (PGE2) secretion level in MSCs [23], which is important for their migration [24] and immunosuppressive activities [25]. Therefore, the development of alternative isolation procedure without G-CSF priming may provide advantages to further clinical applications of PB-MSCs.

Several studies had demonstrated to isolate PB-MSCs with growth factor free method [26–28], while we are the first to establish a growth factor and gradient isolation free protocol. The incubation of PB is required for collecting MSCs secretome in plasma, which has been reported to enhance the stemness and proliferation of MSCs [22, 29, 30]. Glycerin was performed as a supplement for energy sources [31, 32], while preincubation of glycerin with MSCs maintained MSCs survival (Figure S1), marker expression (Figure 4), and stemness of PB-MSCs (Figure S2). It has been demonstrated that after human bone marrow aspirates followed with Ficoll isolation, around 40%–60% of CD105^+^ cells, which are considered as MSCs, will be lost during the procedure [21]. To avoid cell loss, we constructed a culture and isolation medium with ascorbic acid, N-Acetyl-L-cysteine and human platelet lysate. Ascorbic acid has been reported to promote MSCs extracellular matrix formation, which can further modulate stem cell proliferation, self-renewal, and cell fate decisions [23, 33]. N-acetyl-L-cysteine, an antioxidant reagent, can act as a reactive oxygen species (ROS) scavenger to prevent cell cycle arrestment and apoptosis of MSCs and maintain MSCs cell differentiation abilities [24]. Human platelet lysate is a nutrient reagent and contains abundant growth factors and cytokines that promotes MSCs growth [25]. In addition, it also serves as a safe alternative to FBS, which provides a xeno-free in vitro culture system for MSCs expansion [34]. Therefore, we believe that components within our isolation and culture medium can fulfill the criteria for obtaining PB-MSCs.

A previous study demonstrated that MSCs were isolated from one out of six individual PBMCs without growth factors, while they isolated cells with density gradient and cultured with fetus bovine serum [27]. Even isolating MSCs with fibrin microbeads in G-CSF-mobilized PB, MSCs were successfully isolated from eight out of 11 individuals [18], suggesting that the isolation rate is correlated with scanty amount of MSCs within PB. With our isolation procedure, it is noteworthy that attach cell numbers, phenotypes and CT is needed to be carefully monitored. We observed two types of attach cells, spindle and sphere cells, whereas only spindle cells can form colony and be detached. The balance between colony formation and cells became fibrotic is also needed to be considered, while sphere cells may accelerate fibrosis and prevent colony formation, since sphere cells are usually advanced in numbers. The identification of sphere cells and that whether sphere cells secrete factors to avoid MSCs attachment, even survival, requires further analysis.

With our isolation procedure, we obtained adherent cells that highly express CD90, CD73, CD105, CD44, and CD166, while <1% of the cells express CD11b, CD14, CD19, CD34, CD45, and HLA-DR. These isolated candidates performed high expression of stemness genes, especially for *NESTIN* and *LGR5* and remained multidifferentiation abilities, since they can successfully differentiate into adipocytes, osteocytes, and chondrocytes. Gene expression results after 14-day differentiation revealed that these cells have greater potential to differentiate into chrondrocytes, while earlier time points are needed to confirm these results. Also, bone formation capacity for PB-MSCs in vivo are also required before further applications, since it had been reported that differentiation outcomes may be different [35]. Compare to other human MSCs, PB-MSCs may duplicate faster in our culture medium than BM-MSCs and adipose MSCs, but slightly slower than umbilical cord MSCs culturing in commercial or noncommercial medium [36]. Also, the PDT of PB-MSCs are similar to WJ-MSCs in passage 3 [37]. However, the fast increase of PDT after seven passages raise our concern of further application of PB-MSCs.

MSCs are known to encompass immunomodulatory properties with different types of immune cells which contribute to the activation of innate and adaptive immune responses [38]. For example, MSCs reprogram monocytes and macrophages toward M2 phenotype through direct cell-cell contact or paracrine effects [39]. In addition, nature killer cells activities can be modulated by MSCs secretome to prevent over-activation or enhance activation receptor expression [40], while MSC-derived PGE2 interfere PB monocytes to mature into dendritic cells [41]. Like mentioned, the suppression properties of MSCs on activated T cells has been consider as an immunomodulatory function index for isolated MSCs [42]. We examined the duplication of T cells with cell proliferation dye after CD3 and CD28 labeled MACSiBead activation. The proliferation rate of T cells was dose-dependently reduced while being cocultured with isolated MSCs. IL-2 cytokine acts as a growth and survival factor and induces T cell proliferation [43], while IFN-γ enhances clonal expansion and survival through increasing the expression of protein synthesis kinases [44]. PB-MSCs isolated from our procedure significantly suppress both cytokines, which indicates that these MSCs are functional immune-regulators. Possible candidates can further be evaluated to clarify its suppressive mechanism, including paracrine activity, extracellular vesicles, direct cell contact, mitochondria transfer, or MSC differentiation and integration. Finally, by following the International Council for Harmonisation of Technical Requirements for Registration of Pharmaceuticals for Human Use (ICH) instructions, the immunosuppressive quality of MSCs can be qualified by mixed lymphocyte reaction assay [10] to take PB-MSCs into consideration for therapeutic usage. In conclusion, we have provided an alternative method for isolating PB-MSCs that does not require density gradient or growth factor administration. This approach reduces concerns regarding cell loss and is anticipated to facilitate the therapeutic application of PB-MSC-related products.

## Data Availability

The data supporting the findings of this study are available by requesting from the corresponding author.

## Supporting Information

Supporting Information Figure S1. Glycerin pretreatment does not affect PB-MSCs cell viability. PB-MSCs were incubated in a glycerin-containing culture medium or a control medium for 3 h, after which they were transferred to a 12-well plate for an additional 72 h of culture. Following this culture period, a CCK-8 assay was performed and the results were normalized to the control group to verify cell viability. The results are presented as histograms and expressed as mean ± SEM (*n* = 4). Figure S2: Stemness-related genes are expressed in isolated PB-MSCs. Stemness gene expression in cultured cells were analyzed in isolated PB-MSCs after two passages (P3). The expressions of CXCR4, LGR5, NANOG, and NESTIN are quantified and all expressions are normalized with HLA-β2 m for both isolated cells. Results are demonstrated by histogram. Table S1: Primers for stemness, adipogenesis, osteogenesis, and chrondrogenesis. The list of primers used for determination of expression of stemness, adipogenesis, osteogenesis, and chrondrogenesis marker transcripts, which were analyzed by quantitative real-time PCR using SYBR Green Master Mix and CFX Connect Real-Time PCR system. Table S2: Expression of differentiation marker transcripts in undifferentiated cells and differentiated cells. Isolated PB-MSCs were differentiated at Passages 4 and 5 with differentiation medium or differentiation kits. After differentiation, gene expressions against different differentiation targets were analyzed. The expressions of *PPARG*, *FABP4*, and *APOE* represent adipocyte differentiation potential; *RUNX2*, *TIMP3*, and *SPP1* represent osteocyte differentiation potential; *SOX9*, *ACAN*, *COMP*, and *COL11A1* represent chondrocyte differentiation potential. All data sets were normalized with *HLA-B2M* and fold changes were calculated by the comparison between undifferentiated cells for both isolated PB-MSCs.
