# Supplementary material for: Isolate Circulating Mesenchymal Stromal Cells Without Growth Factor Administration and Using Density Gradient
Source: Stem Cells Int. 2025 Jun 19;2025:5545892. doi: 10.1155/sci/5545892 (PMC12202064; doi:10.1155/sci/5545892)
Supplement: Supporting Information — Figure S1. Glycerin pretreatment does not affect PB-MSCs cell viability. PB-MSCs were incubated in a glycerin-containing culture medium or a control medium for 3 h, after which they were transferred to a 12-well plate for an additional 72 h of culture. Following this culture period, a CCK-8 assay was performed and the results were normalized to the control group to verify cell viability. The results are presented as histograms and expressed as mean ± SEM (n = 4). Figure S2: Stemness-related genes are expressed in isolated PB-MSCs. Stemness gene expression in cultured cells were analyzed in isolated PB-MSCs after two passages (P3). The expressions of CXCR4, LGR5, NANOG, and NESTIN are quantified and all expressions are normalized with HLA-β2 m for both isolated cells. Results are demonstrated by histogram. Table S1: Primers for stemness, adipogenesis, osteogenesis, and chrondrogenesis. The list of primers used for determination of expression of stemness, adipogenesis, osteogenesis, and chrondrogenesis marker transcripts, which were analyzed by quantitative real-time PCR using SYBR Green Master Mix and CFX Connect Real-Time PCR system. Table S2: Expression of differentiation marker transcripts in undifferentiated cells and differentiated cells. Isolated PB-MSCs were differentiated at Passages 4 and 5 with differentiation medium or differentiation kits. After differentiation, gene expressions against different differentiation targets were analyzed. The expressions of PPARG, FABP4, and APOE represent adipocyte differentiation potential; RUNX2, TIMP3, and SPP1 represent osteocyte differentiation potential; SOX9, ACAN, COMP, and COL11A1 represent chondrocyte differentiation potential. All data sets were normalized with HLA-B2M and fold changes were calculated by the comparison between undifferentiated cells for both isolated PB-MSCs. [file 5545892.f1.docx]

**Supplement materials**

**
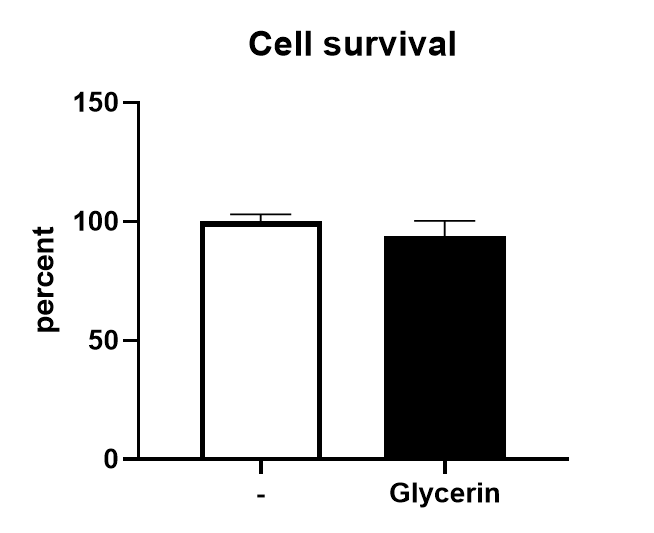
**

**Supplement Figure 1. Glycerin pre-treatment does not affect PB-MSCs cell viability**

PB-MSCs were incubated in a glycerin-containing culture medium or a control medium for 3 hours, after which they were transferred to a 12-well plate for an additional 72 hours of culture. Following this culture period, a CCK-8 assay was performed, and the results were normalized to the control group to verify cell viability. The results are presented as histograms and expressed as mean ± SEM (n=4).


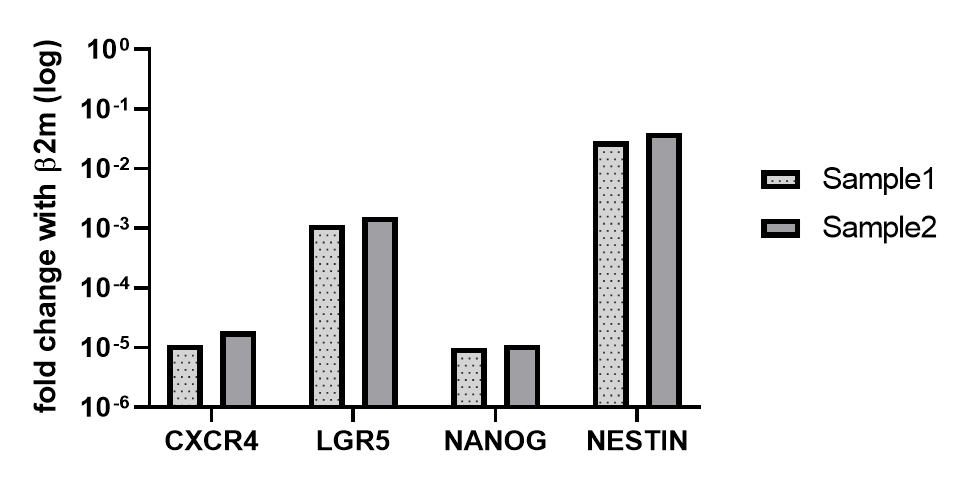


**Supplement Figure 2. Stemness-related genes are expressed in isolated PB-MSCs**

Stemness gene expression in cultured cells were analyzed in isolated PB-MSCs after two passages (P3). The expression of *CXCR4, LGR5, NANOG,* and *NESTIN* are quantified and all expressions are normalized with HLA-β2m for both isolated cells. Results are demonstrated by histogram.

|  | Forward primer | Reverse primer |
| --- | --- | --- |
| h*B2M* | 5’-AGCAGCATCATGGAGGTTTGA-3’ | 5’-TCAAACATGGAGACAGCACTCA-3’ |
| h*CXCR4* | 5’-CCACTGTTGTCTGAACCCCA-3’ | 5’-TGTCCACCTCGCTTTCCTTT-3’ |
| h*LGR5* | 5’-TGAACACCTGCTTGATGGCT-3’ | 5’-TGCTGCGATGACCCCAATTA-3’ |
| h*NESTIN* | 5’-AGTGATGCCCCTTCACCTTG-3’ | 5’-GCTCGCTCTCTACTTTCCCC-3’ |
| h*NANOG* | 5’-AAGAGGTGGCAGAAAAACAACT-3’ | 5’-TCCCTGGTGGTAGGAAGAGTAA-3’ |
| h*PPARG* | 5’-GACAGGAAAGACAACAGACAAATC -3’ | 5’-GGGGTGATGTGTTTGAACTTG -3’ |
| h*APOE* | 5’-AACTGGAGGAACAACTGACCC-3’ | 5’-CGCACACGTCCTCCATGTC-3’ |
| h*FABP4* | 5’-AACTGGTGGTGGAATGCGT-3’ | 5’-GGTCAACGTCCCTTGGCTTA-3’ |
| h*RUNX2* | 5’-GCGGTGCAAACTTTCTCCAG-3’ | 5’-TGCTTGCAGCCTTAAATGACTC-3’ |
| h*TIMP3* | 5’-CACTGGGGAAAGCCTGAGTT-3’ | 5’-GCCAACCCCCACTCTGTAAA-3’ |
| h*SPP1*  (Osteopontin) | 5’-CAACAAATACCCAGATGCTGTGGC-3’ | 5’-GACTTACTTGGAAGGGTCTGTGGG-3’ |
| h*SOX9* | 5’-AGGAAGTCGGTGAAGAACGGG-3’ | 5’-AGCGCCTTGAAGATGGCGT-3’ |
| h*ACAN* | 5’-AACGCAGACTACAGAAGCGG-3’ | 5’AGCGACAAGAAGAGGACACC3’ |
| h*COMP* | 5’-CCGAGTCCGCTGTATCAACA-3’ | 5’-TATGTTGCCCGGTCTCACAC-3’ |
| h*COL11A1* | 5’-CCGTTCCGTTATGGTGGTGA-3’ | 5’-CAATCCGAGCCTGCTGAAGA-3’ |

**Supplement Table 1. Primers for stemness, adipogenesis, osteogenesis and chrondrogenesis**

The list of primers used for determination of expression of stemness, adipogenesis, osteogenesis and chrondrogenesis marker transcripts, which were analyzed by quantitative real-time PCR using SYBR Green Master Mix and CFX Connect Real-Time PCR system.

| **Adipogenesis** | | | | |
| --- | --- | --- | --- | --- |
| **Gene** | ΔΔct | | Fold change | |
|  | Sample 1 | Sample 2 | Sample 1 | Sample 2 |
| *PPARG* | -5.93 | -5.73 | 60.97 | 52.89 |
| *FABP4* | -0.81 | -1.08 | 1.75 | 2.114 |
| *APOE* | -0.69 | -0.615 | 1.61 | 1.53 |
| **Osteogenesis** | | | | |
| **Gene** | ΔΔct | | Fold change | |
|  | Sample 1 | Sample 2 | Sample 1 | Sample 2 |
| *RUNX2* | -14.03 | -11.82 | 16728.26 | 3603.04 |
| *TIMP3* | -0.69 | -0.60 | 1.61 | 1.51 |
| *SPP1 (OPN)* | -2.32 | -1.48 | 4.99 | 2.78 |
| **Chondrogenesis** | | | | |
| **Gene** | ΔΔct | | Fold change | |
|  | Sample 1 | Sample 2 | Sample 1 | Sample 2 |
| *SOX9* | -3.99 | -2.57 | 15.89 | 5.94 |
| *ACAN* | -1.84 | -3.06 | 3.58 | 8.34 |
| *COMP* | -10.34 | -9.2 | 1296.13 | 588.13 |
| *COL11A1* | -5.81 | -6.59 | 56.1 | 96.36 |

**Supplement Table 2. Expression of differentiation marker transcripts in undifferentiated cells and differentiated cells**

Isolated PB-MSCs were differentiated at passage 4-5 with differentiation medium or differentiation kits. After differentiation, gene expressions against different differentiation targets were analyzed. The expression of *PPARG, FABP4* and *APOE* represent adipocyte differentiation potential; *RUNX2, TIMP3* and *SPP1* represent osteocyte differentiation potential; *SOX9, ACAN, COMP* and *COL11A1* represent chondrocyte differentiation potential. All data sets were normalized with *HLA-B2M* and fold changes were calculated by the comparison between undifferentiated cells for both isolated PB-MSCs.
